# Clinical Performance Evaluation of an Artificial Intelligence–Based Tool for Predicting the Presence of Obstructive Coronary Artery Disease: Protocol for a Cohort Observational Study

**DOI:** 10.2196/67697

**Published:** 2025-09-25

**Authors:** Georgios Rampidis, Evangelos Logaras, Athanasios Samaras, Emmanouil S Rigas, Ilias Kyparissidis-Kokkinidis, Styliana Siakopoulou, Panagiotis-Emmanouil Kartsidis, Konstantinos Kouskouras, George Giannakoulas, Panagiotis Bamidis, Antonios Billis

**Affiliations:** 1 First Department of Cardiology University General Hospital of Thessaloniki AHEPA Thessaloniki Greece; 2 Cardiac Imaging Unit Diagnostic Center “PANAGIA” Veroia Greece; 3 Lab of Medical Physics and Digital Innovation Aristotle University of Thessaloniki Thessaloniki Greece; 4 First Department of Radiology University General Hospital of Thessaloniki AHEPA Thessaloniki Greece

**Keywords:** coronary computed tomography angiography, CCTA, CAD, coronary artery disease, cardiology, stenosis prediction, AI, protocol, ML, machine learning, artificial intelligence

## Abstract

**Background:**

A significant number of individuals undergoing coronary computed tomography angiography (CCTA) for suspected (CAD) have nonobstructive or no CAD. There is a need for clinically proven models that can predict the pretest probability of stable CAD and help to identify low-risk individuals. Optimizing patient stratification is of paramount importance to improve diagnostic yield and cost-effectiveness.

**Objective:**

We aimed to determine whether each patient needs to undergo CCTA because of suspected CAD. The main objective of this study is to evaluate the clinical performance of an artificial intelligence (AI)-based tool in predicting significant coronary artery stenosis (>50%), as well as its utility by medical professionals.

**Methods:**

Data for this study have been acquired from 750 participants as part of routine clinical practice in AHEPA (American Hellenic Educational Progressive Association) General Hospital of Thessaloniki. The dataset has several features, including demographics (eg, age, gender), medical history (eg, diabetes mellitus, arterial hypertension), and clinical variables (eg, creatinine, epicardial fat volume). At least 2 expert cardiologists and 2 expert radiologists are involved in this study, who provide the ground truth. A trained AI-based model embedded in an easy-to-use and user-friendly web application is implemented in practice. Several AI algorithms are being examined, and the model found to perform best so far is the Optimized Voting model, which is a combination of the best performing iterations of random forest and extreme gradient boosting. The performance metrics that are being used are accuracy, precision, recall, *F*_1_-score, area under the receiver operating characteristic curve, and area under the precision-recall curve.

**Results:**

Recruitment for this study began in July 2023. Data collection, development, training, and deployment of the AI web tool were completed by May 2024. In total, data from 500 individuals were collected for training and internal validation, while the best performing model was validated externally in another 250 individuals. For training and internal validation, the dataset was split into 70% for training and 20% for validation and 10% for testing. Currently, the best performing model achieves an accuracy of approximately 82% in successfully predicting stenosis greater than 50%. Additionally, an explainable AI algorithm is used to provide explanations in relation to the decisions made aiming to increase the trust of the clinicians in the tool.

**Conclusions:**

The proposed study represents a novel approach of a web-based AI-driven solution with explainability features for optimizing patient stratification with the goal of improving diagnostic yield and cost-effectiveness of CCTA utilization within the context of cardiology clinical practice.

**International Registered Report Identifier (IRRID):**

DERR1-10.2196/67697

## Introduction

### Background

A conventional routine in clinical practice over the years has been to employ validated diagnostic models of the pretest probability (PTP) of stable, albeit obstructive, coronary artery disease (CAD) [1] in order to direct downstream testing. After the first screening, adults with suspected CAD with existing symptoms and low-to-intermediate PTP undergo coronary computed tomography angiography (CCTA) [2], including calcium scoring.

Most existing models have modest performance (with remarkable overestimation of risk in certain subgroups such as women), while very few studies have data regarding the effect of PTP-based models on clinical decision-making regarding further testing or patient outcomes [3]. The European Society of Cardiology clinical practice guidelines for the management of stable chest pain congruently suggest CCTA as a first-line diagnostic option in symptomatic cases, which are thought to have a low-to-intermediate pretest odds of being diagnosed with obstructive CAD [4]. Specifically, suspected cases during their checkup routine or individuals with suspected CAD undergo a computed tomography (CT) scan with coronary artery calcium scoring, along with other examinations and finally CCTA to obtain the final diagnosis. Results evaluation (laboratory results, CCTA analysis, coronary artery geometric features, coronary artery calcium score, etc) is based exclusively on the treating cardiologist’s experience. In case of suspected coronary heart disease, patients might receive further, more extensive, but possibly unnecessary examinations such as invasive coronary angiography or single-photon emission CT, which last up longer tying up hospital resources, increase patients’ radiation exposure, and may have adverse effects.

However, in day-to-day clinical practice, a significant number of individuals undergoing CCTA have minimal or no CAD. As a direct consequence of the expanding use of CCTA, there is a growing interest within the medical community regarding ways to optimize patient stratification with the goal of improving diagnostic yield and the cost-effectiveness of CCTA utilization within the context of clinical practice. Hence, there is a need for clinically validated models that can predict the PTP of stable CAD and as a result function as gatekeepers to identify low-risk individuals who are unlikely to have obstructive CAD and unlikely to need further diagnostic testing, including CCTA. Such models can be based on powerful machine learning (ML) algorithms that have already proven effective in several health care–related problems [5].

### Objectives

In the context of this study, an artificial intelligence (AI)-based predictive model integrated into a web application was developed. Patients’ clinical results and variables extracted from the screening procedure in combination with demographics, medical history, social history, and other medical data were entered into the application, which indicated, with an accuracy of at least 70%, whether each individual has over 50% stenosis, which leads to the need to undergo CCTA, including calcium scoring, because of suspected CAD. The specific objectives of the study are listed below.

#### Primary Objective

The primary objective of this study was to evaluate the clinical performance of the ΑΙ-based tool developed, which was measured as the percentage of successful predictions, confirmed by experienced medical staff.

#### Secondary Objective

The secondary objective was to evaluate the professionals’ satisfaction regarding the AI-based tool based on the feedback after its use.

## Methods

### Study Environment

The data for this study were collected from individuals visiting the cardiology outpatient clinic of AHEPA (American Hellenic Educational Progressive Association) General Hospital of Thessaloniki, Greece.

### Framework

The main portion of this study was conducted during the pilot phase of the “Hospital Smart development based on AI - HosmartAI” project [6], which falls under the Horizon 2020 initiative and has received funding from the European Commission (grant 101016834). The HosmartAI project ran from January 2021 to May 2024 and aimed to transform health care by integrating AI and robotics. It brought together 24 partners from 12 countries to develop the HosmartAI Hub, an open platform supporting AI-driven solutions through tools like a marketplace and benchmarking functionalities. Through 8 pilots across various medical domains, the project improved diagnosis, treatment, and care delivery, fostering a more efficient and sustainable health care ecosystem in Europe.

### Participants

Data were acquired from 750 individuals (500 for algorithm training and testing and 250 during the external validation) as part of routine clinical practice in AHEPA General Hospital of Thessaloniki. At least 2 expert cardiologists and 2 expert radiologists were involved in this study, who provided the ground truth.

#### Inclusion Criteria

Individuals eligible for data collection met the following criteria: (1) age ≥18 years and (2) individuals experiencing chest pain who underwent CCTA to exclude obstructive CAD.

#### Exclusion Criteria

Individuals were excluded if they refused to give written consent to participate in the study.

### Data Collection Plan

Study participants visiting the AHEPA General Hospital of Thessaloniki for examination and deemed eligible for data collection by the collaborating medical team were invited to participate in this study. Enrollment in this study and data acquisition were performed on the same day. Study participants first reviewed the study information sheet and then gave informed consent. Once consented, they were assigned a coded ID number. Afterwards, basic demographics, medical history, social history, prior laboratory results, and other medical variables formerly indicated by the medical staff were recorded linked with the participant’s ID. CCTA outcome (actual scan file and analysis variables) acquisition followed based on standard clinical practice. At the end of the study, data on the satisfaction of the participating health care professionals were also collected.

### Ethical Considerations

During the consent process, study participants are duly informed about their rights concerning the collected data as per the guidelines laid out in the General Data Protection Regulation and Greek law (4624/2019). Each participant provided written consent after gaining a comprehensive understanding of the procedures involved. The investigator or a member of the study staff thoroughly explained the consent forms and procedures, including the study's objectives, methodologies, potential benefits, and risks. Before enrolling into the study, the individuals signed these consent forms, indicating their voluntary participation. They were explicitly informed that they retain the option to withdraw from the study at any time without affecting their treatment care. Ample time was provided for participants to review the study information and consent form and seek clarifications, if needed. Additionally, upon signing the informed consent document, each participant received a copy for their records. Data were anonymized before being used. No compensation was given to the participants. This study was submitted and approved by the Research Ethics and Conduct Committee of the Aristotle University of Thessaloniki (94517/2022).

### Data Management

All CCTAs were performed at the AHEPA General Hospital of Thessaloniki by experienced cardiologists using the hospital's equipment. Study personnel of the AHEPA General Hospital of Thessaloniki were responsible for the proper management of data stored at the hospital. All data, including the professionals’ satisfaction results, were stored in a dedicated, password-protected computer of the AHEPA General Hospital of Thessaloniki, with limited access. Study hard copy documents, including signed informed consent documents, were kept in locked cabinets with limited access.

### Access to Data

Investigators from the First Department of Cardiology, the Laboratory of Radiology and Radiodiagnostics, and the Laboratory of Medical Physics and Digital Innovation of AUTH who are authorized by the principal investigator were given access to the acquired, deidentified data sets, to ensure participant confidentiality. In case of data transfer over a computer network, this took place through safe processes, and data were stored in secure digital structures with limited access.

In the context of research reproducibility, completely deidentified datasets could be published to one or more data repositories for sharing purposes, accompanied by an appropriate license that were agreed among the First Department of Cardiology, the Laboratory of Radiology and Radiodiagnostics, and the Laboratory of Medical Physics and Digital Innovation of AUTH, in accordance with FAIR (Findable, Accessible, Interoperable, and Reusable) principles.

Study participants have access to the data they contributed to the study, in compliance with the respective mandate of the General Data Protection Regulation. Participants are also able to request for their data to be deleted. Instructions for requesting, receiving, or deleting data are included in the study information sheet. The professionals’ satisfaction results were collected anonymously at first hand.

### Data Analysis

In the process of data analysis, ML algorithms were studied, reviewed, and tested for highest accuracy. Specifically, the random forest [7], extreme gradient boosting (XGBoost) [8], Bagging [9], and Optimized Voting algorithms were considered and tested. Random forest is an ensemble method that constructs numerous decision trees during training and merges their outcomes to enhance accuracy and reduce overfitting. XGBoost is an advanced gradient boosting algorithm known for its efficiency and performance on structured data, capable of handling missing values and reducing overfitting. Bagging is an ensemble technique that improves the stability and accuracy of ML algorithms by training multiple models on different subsets of the data and combining their predictions. Optimized Voting is a method that aggregates the predictions of several models to make a final decision, aiming to achieve superior performance compared to any single model. Hyperparameters for each model were fine-tuned using Bayesian optimization [10]. Moreover, given that interpretability is crucial in clinical applications to allow health care professionals to understand the reasoning behind model predictions, SHAP (Shapley Additive Explanations) [11] was employed for this purpose.

Training, validation, and testing of these methods were achieved using the dataset collected in the study. Stenosis >50%, that is, if there is more than 50% stenosis and hence confirming that there is the need for the more invasive CCTA procedure, was used as the predictor variable. Stenosis >50% is the point where further actions are necessary [12]. Regarding the whole process, standard practices were followed, that is, a 3-part partition of the data was performed: 500 individuals (100%) were classified into a training (70%), validation (20%), and testing (10%) set. An additional set of 250 individuals was exclusively used for external validation. Once each algorithm was trained and optimized through the training and validation set, they were applied to the testing set to determine the final accuracy of each method. Six standard evaluation metrics were used to assess the predictions of all the classifiers. These include area under the receiver operating characteristic curve, area under the precision-recall curve, accuracy, precision, recall, and *F*_1_-score. The area under the receiver operating characteristic curve reflects the best balance between sensitivity and specificity, whereas the area under the precision-recall curve shows the balance between precision and recall. Ten-fold cross-validation was used to avoid overfitting. Finally, the algorithm with the highest prediction accuracy was selected for use in the system.

Additionally, to collect the opinions of health care professionals, a set of cardiologists with different experience levels was selected. They were provided with the web application and specific instructions on how to use it, and they completed 2 questionnaires. Specifically, the usefulness, satisfaction, and ease of use (USE) questionnaire [13] was employed to assess perceived usefulness, while the System Usability Scale (SUS) questionnaire [14] was used to evaluate perceived usability. The replies of all the participants were collected, and descriptive statistics were calculated based on them.

### Implementation of the ML Algorithms and the Web Tool

In parallel with the development of the predictive model, a comprehensive web application was also developed. This application integrates multiple technology platforms, including Keycloak, Angular, HAPI FHIR, Python, and Nginx, each contributing essential features to create a cohesive system that meets the set requirements. The architecture is divided into several modules, each addressing specific functionalities within the system. These modules include User Authentication and Authorization, the front-end application for data input and management, the Health Care Data Management Backend for secure data storage and retrieval, the Predictive Analytics Application for generating outcome predictions, and Networking and Load Balancing to ensure optimal performance and resource allocation. The integration of these components results in a user-friendly, secure, and performance-optimized application. This application provides valuable insights to health care providers, supporting data-driven decisions that can lead to more effective treatments and improved patient outcomes.

## Results

The study began with the submission of the study protocol to Aristotle University of Thessaloniki ethics committee in March 2022, followed by approval in April 2022. Data collection started in May 2022 and concluded in June 2023, with a total of 500 patients enrolled. These data were used for the development and testing of ML models designed to predict stenosis greater than 50%. The development of the AI algorithms was finalized in March 2024. Additionally, an explainable AI component was developed to clarify the decisions made by the algorithms and enhance their acceptability among health care professionals. Both the ML models and the explainable AI component were integrated into a web-based tool. This tool was subsequently evaluated externally by using data from additional 250 cases. The data from this external validation are currently being analyzed. The best performing model so far achieves an accuracy of approximately 82% in successfully predicting stenosis greater than 50%. Moreover, the web tool was assessed by several health care professionals. Specifically, a total of 12 cardiologists participated by responding to the USE and SUS questionnaires, with the sample comprising 10 male (83%) and 2 female (17%) physicians. Concerning usefulness, over 60% (8/12) of the participants responded positively to each question in this category, confirming the application’s utility in daily medical practice. Regarding usability, 25% (3/12) of the participants rated the application as D (poor), another 25% (3/12) rated it as B (good), and 33% (4/12) rated it as A (excellent). The average score on the SUS questionnaire was 72.08, corresponding to a grade of B (good).

## Discussion

### Comparison to Prior Work

Several attempts have been made to create systems that accurately detect and classify coronary artery plaque and stenosis. For example, Zreik et al [15] suggest a noninvasive, automatic approach for identifying patients in need of invasive coronary angiography. To do so, they employed ML techniques, including a support vector machine classifier, to analyze coronary arteries in cardiac CCTA images with promising results. In a similar vein, a study by Han et al [16] investigated the feasibility of using deep learning algorithms to analyze CCTA images in patients with coronary artery stenosis. To create autoreconstructed CCTA images based on a sequence of 2D CT scans, they designed a CCTA reconstruction pipeline by using deep learning and transfer learning methodologies;150 patients who had CCTA and digital subtraction angiography were studied retrospectively. Their results show that when compared to conventional approaches, AI significantly lowers the time required for postprocessing and diagnosis.

Furthermore, for diagnosing functionally obstructive CAD, Coenen et al [17] compared the diagnostic performance of ML-based CT-fractional flow reserve (CT-FFR) to CTA and computational fluid dynamics–based CT-FFR; 351 individuals were studied at 5 facilities throughout Europe, Asia, and the United States, with 525 vessels undergoing invasive FFR comparison. On the CTA data, ML-based and computational fluid dynamics–based CT-FFR were conducted, and diagnostic performance was assessed using invasive FFR as a reference. Diagnostic accuracy increased from 58% with CTA to 78% by using ML-based CT-FFR on a per-vessel basis.

Cho et al [18] used a sample of 1501 patients with stable and unstable angina aiming to categorize lesions as having an FFR of 0.80 by using an angiography-based supervised ML system that was developed. Specifically, XGBoost employed 24 computed angiographic characteristics based on the diameter plot, as well as 4 clinical features (age, sex, body surface area, and involve segment). We used 2000 bootstrap iterations to train and evaluate the model separately. A total of 79 patients were used for external validation. Using all 28 characteristics, the ML model predicted an FFR of 0.80 in the 1204 training samples, with an overall diagnostic accuracy of 78% (SD 4%). At the same time, Han et al [19] aimed to determine the incremental value of resting myocardial CT perfusion over coronary stenosis in predicting ischemia by using an ML-based computational system. They used CCTA and invasive FFR that were performed on 252 individuals. Resting CT perfusion analysis was conducted utilizing supervised ML with a gradient boosting classifier. Their results show that resting CT perfusion data obtained by ML approaches may increase the prediction usefulness of severe ischemia over coronary stenosis.

Yang et al [20] focused on aortic stenosis classification, and they used cardio-mechanical signals that were collected with wearable inertial sensors. Data were collected from 21 patients with aortic stenosis and from 13 participants with no stenosis. Features were generated using a continuous wavelet transform and reduced based on an Elastic Net framework. The performance of different ML techniques was compared. Additionally, a 2D convolutional neural network was constructed using a custom-built architecture and a convolutional neural network trained using transfer learning on MobileNet. Their findings demonstrate the framework's usefulness in terms of feature selection and categorization. Additionally, they provide a strong potential for using deep learning technologies in the categorization of aortic stenosis.

Lin et al [21] conducted a multinational, multicenter study that included 9 cohorts of patients who had CCTA at 11 locations and were randomly allocated to training and test sets. A unique convolutional neural network trained using deep learning was used to segregate coronary plaque in 921 individuals (5045 lesions). The deep learning network was subsequently applied to an independent test set consisting of 175 patients (1081 lesions) with external validation and 50 patients (84 lesions) with intravascular ultrasonography within 1 month after CCTA. They assessed the prognostic value of deep learning–based plaque measurements for fatal or nonfatal myocardial infarction (the primary outcome) in 1611 patients enrolled in the prospective SCOT-HEART trial by using multivariable Cox regression analysis with adjustment for the ASSIGN clinical risk score. Expert readers and intravascular ultrasonography agree well with the deep learning system's estimations of plaque volume and stenosis severity from CCTA, which may be predictive for future myocardial infarction.

Finally, the purpose of a retrospective multicenter research conducted by Xu et al [22] was to determine the diagnostic accuracy and generalizability of a well-established fully automated deep learning method for identifying coronary stenosis based on CCTA. A total of 527 patients with suspected CAD who underwent CCTA and invasive coronary angiography were recruited. The diagnostic accuracy of the deep learning algorithm in detecting 50% stenosis was compared to that of professional readers by using invasive coronary angiography as a standard reference. According to the authors, the deep learning algorithm had good generalizability and time efficiency and performed similarly to expert readers on CCTA in terms of CAD diagnosis.

Compared to previous works, our study leverages ML for the prediction of significant coronary artery stenosis. The developed ML model was trained and tested using a real dataset, and it was further validated in a real-world clinical setting. Notably, our model does not rely on contrast-enhanced CCTA, thereby reducing patient exposure to contrast agents and associated risks, lowering costs, and making the screening process safer and more accessible for a broader range of patients. Although the results are still being analyzed, they appear promising. Furthermore, the ML model was integrated in a web-based application designed for routine clinical use, enhancing both usability and transparency. Finally, our approach incorporates explainable AI techniques, enabling clear and interpretable predictions, which is an essential factor for clinical acceptance and trust.

### Limitations

When interpreting the findings of our study, a number of restrictions should be considered. First, all the information used in this study came from the AHEPA General Hospital in Thessaloniki, Greece. Thus, the results might not apply to other populations or clinical settings, particularly those with various demographic traits or health care systems. Second, the small sample size of this study might make it difficult for the AI tool to reliably predict CAD in a wide range of patient populations. The model's performance across various subgroups will be evaluated in future studies with larger and more diverse samples. Third, because the study depends on the accuracy of the data entered into the AI tool, any errors in data entry could have an impact on the outcomes. Fourth, as the AI tool is used in the real world, its predictive accuracy will need to be continually assessed and adjusted based on ongoing performance metrics. Finally, it is crucial to continuously monitor and address the ethical issues relating to data privacy, security, and patients' capacity to comprehend and consent to the use of AI in their care.

### Conclusions

The goal of this pilot study was to determine whether an AI-based predictive model can help individuals with suspected CAD avoid unnecessary diagnostic tests. The proposed model was intended to predict whether a patient has more than 50% stenosis, which would call for additional testing, using data from patient screenings along with other pertinent information (demographics, medical history, etc). This strategy fits with a growing trend in health care that uses ML algorithms to enhance clinical practice and patient outcomes. Our research expands on earlier studies that classified and identified coronary artery plaque and stenosis by using ML techniques. Although encouraging, these studies have brought attention to the need for larger and more varied datasets to increase the predictive precision of the ML models. Our study also considers the insightful comments provided by the clinical professionals who use the AI tool, which helps to make it practical for use in real-world scenarios. The developed AI tool has the potential to speed up diagnosis, increase clinical satisfaction, and improve clinical performance in the diagnosis of CAD. For the validation of the efficacy of this AI tool in a real-world clinical setting, the findings of this pilot study are essential.

## Appendix

Multimedia Appendix 1 Peer review report by Horizon 2020 - Research and Innovation Framework Programme, European Commission.

## Abbreviations

AHEPA: American Hellenic Educational Progressive Association
AI: artificial intelligence
CAD: coronary artery disease
CCTA: coronary computed tomography angiography
CT: computed tomography
CT-FFR: computed tomography-fractional flow reserve
FAIR: Findable, Accessible, Interoperable, and Reusable
FFR: fractional flow reserve
ML: machine learning
PTP: pretest probability
SHAP: Shapley Additive Explanations
SUS: System Usability Scale
USE: usefulness, satisfaction, and ease of use
XGBoost: extreme gradient boosting

## Data Availability

The datasets generated during this study will be available from the corresponding author on reasonable request.
